# Trifocal versus Pentafocal bone transport in segmental tibial defects: a matched comparative analysis for posttraumatic osteomyelitis treatment

**DOI:** 10.1186/s12891-024-07507-w

**Published:** 2024-05-15

**Authors:** Yimurang Hamiti, Patiman Abudureyimu, Gang Lyu, Aihemaitijiang Yusufu, Maimaiaili Yushan

**Affiliations:** 1 Department of Microrepair and Reconstructive Surgery, The First Affiliated Hospital of Xinjiang Medical University, Urumqi, Xinjiang P. R. China; 2 Imaging Center of the First Affiliated Hospital of Xinjiang Medical University, Urumqi, Xinjiang P. R. China; 3 Department of Orthopedic Surgery, The Fourth Affiliated Hospital of Xinjiang Medical University, Traditional Chinese Medicine Hospital of Xinjiang Uyghur Autonomous Region, Urumqi, Xinjiang P. R. China

**Keywords:** Bone transport, Distraction osteogenesis, Ilizarov technique, Tibial defect, Osteomyelitis

## Abstract

**Purpose:**

The objective of this study was to evaluate and compare the effectiveness and clinical results of trifocal bone transport (TBT) and pentafocal bone transport (PBT) in treating distal tibial defects > 6 cm resulting from posttraumatic osteomyelitis, highlighting the potential advantages and challenges of each method.

**Methods:**

A retrospective assessment was conducted on an overall population of 46 eligible patients with distal tibial defects > 6 cm who received treatment between January 2015 and January 2019. Propensity score analysis was used to pair 10 patients who received TBT with 10 patients who received PBT. The outcomes assessed included demographic information, external fixation time (EFT), external fixation index (EFI), bone and functional outcomes assessed using the Association for the Study and Application of the Method of Ilizarov (ASAMI) scoring system, and postoperative complications evaluated using the Paley classification.

**Results:**

The demographic and baseline data of the two groups were comparable. Following radical debridement, the average tibial defect was 7.02 ± 0.68 cm. The mean EFT was significantly shorter in the PBT group (130.9 ± 16.0 days) compared to the TBT group (297.3 ± 14.3 days). Similarly, the EFI was lower in the PBT group (20.67 ± 2.75 days/cm) than in the TBT group (35.86 ± 3.69 days/cm). Both groups exhibited satisfactory postoperative bone and functional results. Pin site infection was the most common complication and the rates were significantly different between the groups, with the PBT group demonstrating a higher incidence.

**Conclusion:**

Both TBT and PBT effectively treat posttraumatic tibial defects greater than 6 cm, with PBT offering more efficient bone regeneration. However, PBT is associated with a higher rate of pin site infections, highlighting the importance of careful management in these complex procedures and emphasizing the need for expert surgical execution and tailored treatment approaches in orthopedic reconstructive surgery.

## Introduction

Orthopedic surgery often addresses critical bone defects, which are gaps in the bone that are unlikely to heal spontaneously due to their size or the disruption of the vascular supply [1]. These defects present a major therapeutic challenge, particularly when they affect the tibia, a bone essential for weight-bearing. Segmental tibial defects, especially those resulting from posttraumatic osteomyelitis, are further complicated by issues such as impaired blood supply, inadequate soft tissue coverage, risk of infection, non-union, and potential malunion. These complications can disturb mechanical alignment and function, exacerbating the situation. Many patients with these defects have sustained them from high-energy trauma, accompanied by additional injuries that may impede early healing [2–4]. In addressing this concern, a range of therapeutic approaches have been recognized as efficacious. Autologous bone grafts, the induced membrane technique, the Ilizarov method (based on distraction osteogenesis principles), and vascularized and nonvascularized fibular grafting techniques are among the methodologies that can be implemented [5–13]. Determining the most appropriate treatment strategy is contingent upon a multitude of crucial considerations, encompassing the defect’s precise site and extent, as well as any additional injuries.

Among the diverse array of techniques utilized, bone transport using the Ilizarov method has emerged as a pioneering advancement, demonstrating promising results in terms of bone healing and restoration of functionality. The Ilizarov method has the ability to simultaneously address various orthopedic challenges, such as the management of limb discrepancies and deformities, whether acquired or congenital, in addition to the treatment of composite bone and soft-tissue defects [2–4, 14, 15]. This technique has been thoroughly validated over time, with a significant body of long-term studies underscoring its efficacy. A notable single-center study spanning 15 to 30 years confirmed that Ilizarov bone transport effectively managed comminuted tibial fractures and deformities and maintained functional outcomes in line with contemporary standards [16]. The technique of multifocal bone transport has been devised and utilized to address the obstacles that arise due to the prolonged duration of external fixation needed for long-distance bone segment transport. These techniques employ multiple osteotomies to facilitate bone regeneration over considerable distances, potentially reducing treatment time and enhancing functional outcomes [17–24].

Specifically, trifocal bone transport (TBT), involving two osteotomy sites, is designed to efficiently manage moderate to large bone defects. The primary advantage of TBT lies in its ability to reduce the duration of treatment and external fixation, thereby diminishing patient discomfort and the risk of infection. However, its complexity increases with the number of transport segments, which requires meticulous surgical precision and postoperative management to avoid complications such as misalignment and incomplete ossification [17–23]. Pentafocal bone transport (PBT), which involves four osteotomy sites, is suitable for larger and more complex defects that require extensive bone regeneration. The advantage of PBT is its enhanced capacity for bone regeneration over larger distances, potentially leading to better structural outcomes. Nonetheless, the increased number of osteotomy sites escalates the procedural complexity and may increase the risk of surgical complications [24]. Both TBT and PBT offer theoretical advantages over traditional methods by potentially minimizing treatment durations and improving functional outcomes, yet they necessitate careful consideration of their increased technical demands and potential for complications.

Despite their increasing application, a comprehensive comparisons between these multifocal techniques are rare in the literature. Such a comparison is crucial, not only for delineating their respective efficacies and complication profiles but also for guiding surgeons toward an optimal choice based on defect characteristics and patient-specific factors. The present study proposes to conduct a matched comparative analysis of TBT versus PBT in the treatment of segmental tibial defects due to posttraumatic osteomyelitis. This comparison aimd to provide insights into optimizing treatment strategies for complex orthopedic reconstructions. To guide our investigation, we hypothesize that the efficacy and complication profiles of multifocal bone transport techniques vary significantly, influencing clinical outcomes in patients with tibial segmental defects. Specifically, we intend to answer the following research question: What are the overall efficacy, safety, and comparative advantages and challenges of trifocal and pentafocal bone transport in the treatment of tibial segmental bone defects caused by posttraumatic osteomyelitis? To the best of our knowledge, this is the first paper that compares the efficacy and clinical outcomes of TBT and PBT in this field.

## Patients and methods

### Study design and patient selection

The present study was a retrospective comparative investigation conducted at a single center. The primary objective was to examine outcomes in patients with segmental distal tibial defects greater than 6 cm following radical debridement due to posttraumatic osteomyelitis. This inclusion criterion was selected because defects of this size are generally not amenable to acute shortening and require more complex reconstructive techniques such as segmental bone transport. Larger defects (> 6 cm) necessitate the use of bone transport techniques to ensure effective bone and soft tissue regeneration. Smaller defects might be managed with less extensive methods, such as acute shortening or vascularized bone grafts, which are insufficient for larger gaps due to their limitations in addressing extensive bone loss. To ensure consistency in surgical outcomes, all procedures were performed by the same surgical team and remained consistent throughout the study period. The study protocol was approved by our institute’s Institutional Ethics Committee. All patients provided written informed consent before participation, and the study protocols were approved by the ethical committee of our institution. All methods were carried out in accordance with relevant guidelines and regulations [IRB No: 20190514-10]. We retrospectively reviewed cases treated between January 2015 and January 2019. Inclusion criteria encompassed patients with distal tibial defects more significant than 6 cm following posttraumatic osteomyelitis, treated via TBT or PBT. Exclusion criteria included patients with tibial defects from other etiologies (e.g., tumor removal), individuals under 18 years of age, or those with insufficient follow-up data. The study ultimately included 46 patients, 36 undergoing TBT and 10 receiving PBT. A minimum follow-up duration of two years postexternal fixator removal was mandated for inclusion.

### Data collection and propensity score matching

Preoperative planning and radiological evaluations were conducted by the senior authors, ensuring a standardized approach. Detailed data, including radiological findings, operative records, and medical histories, were meticulously extracted from medical records. This data extraction and subsequent analysis were performed by three surgeons (Y.H., P.A., and G.L.). Additionally, demographic and baseline information, such as age, gender, injury mechanism, affected side, defect size and location, previous operations, and follow-up duration, was systematically recorded by two other surgeons (A.H. and M.Y.). To ensure comparability between the two groups, we employed Propensity Score Matching (PSM). On the basis of age, gender, affected side, defect size, prior operation time, and follow-up period, patients were matched on a 1:1 form. This matching aimed to minimize confounding factors and bias, thereby enhancing the validity of our comparisons.

### Bone transport technique

Patients were positioned on the operating table, and anesthesia was administered, either continuously epidural or generally, depending on individual patient factors and surgical requirements. The initial step involved thorough radical debridement. This included the removal of all nonvital bone and soft tissues, along with any previous surgical implants. Bone and soft tissue samples were collected for culture and drug susceptibility tests to guide the postoperative antibiotic regimen. Based on our accrued experience with bifocal and trifocal bone transport for large tibial defects, the length of each individual bone segment was maintained at a minimum of 3–4 cm. Following debridement, the external fixator was assembled and installed, as dictated by the preoperative plan. Minimally invasive percutaneous osteotomy was performed at the preselected osteotomy sites using a Gigli saw. TBT involves creating two additional corticotomies apart from the primary defect site, thus forming two active bone regeneration zones. The segments were gradually transported using an external fixator until the defect was bridged. In contrast, the PBT entailed four additional corticotomies, which formed four regeneration zones. The same principles of gradual distraction (proximal to distal) were applied. The process of transporting individual bone segments was initiated following a latency period ranging from 7 to 10 days.

### Outcome evaluation and follow-up protocol

All study participants underwent regular follow-up, scheduled biweekly at our outpatient clinic. This follow-up regimen included radiographic examinations to monitor union progress and assess pain-free mobility. Adjustments to the rate and rhythm of distraction were made based on the radiographic evaluation of each distraction site and patient tolerance. The external fixator was removed following a dynamization period of one month, which commenced once the transferred bone segment reached the docking site and a minimum of three bridging calluses were visible on both anteroposterior and lateral radiographs [25]. The duration from frame application to removal, termed external fixation time (EFT), was recorded in days. Furthermore, the external fixation index (EFI) was calculated by dividing the EFT by the length of the regenerated bone (days/cm). Bone and functional outcomes were evaluated using the Association for the Study and Application of the Method of Ilizarov (ASAMI) score system [17]. Complications encountered during treatment were meticulously recorded and categorized as per the Paley classification, which differentiates between problems, obstacles, and true complications [26]. This classification system, which is essential for standardizing adverse event assessments in deformity correction and lengthening procedures, distinguishes a ‘problem’ as an issue resolved by the end of treatment without surgical intervention, an ‘obstacle’ as a complication resolved surgically by treatment conclusion, and a ‘true complication’ as a persistent issue during the posttreatment period.

### Statistical analysis

Statistical analysis was performed using SPSS software (version 25.0, IBM Corp). Prior to the analysis, the distribution of continuous variables was assessed using the Shapiro-Wilk test. Independent t-tests were made between the two groups for data that followed a normal distribution. The Mann-Whitney U-test was utilized for non-normally distributed continuous or ordinal data. Categorical variables were analyzed using the chi-squared test or Fisher’s exact test. A *P*-value of less than 0.05 was considered statistically significant.

## Results

### Demographic and clinical characteristics

**Fig. 1:**
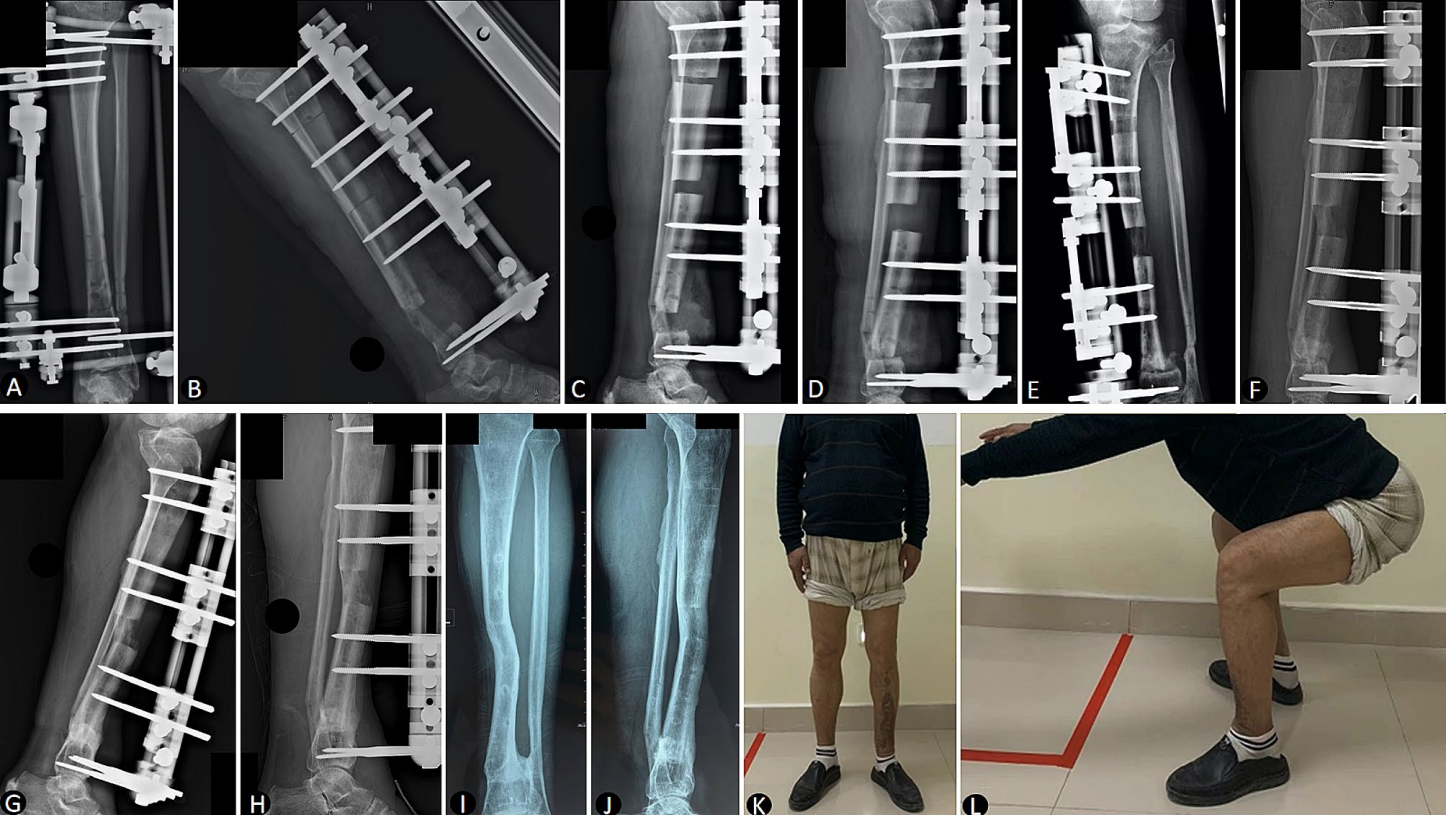
**A** A 52-year-old male patient with posttraumatic osteomyelitis of the left distal tibia. **B** Surgical removal of the infected bone and soft tissue resulted in a 6.3 cm defect, and beginning of trifocal bone transport. **C, D,E** A radiographic series delineating the trifocal bone transport phases, capturing the stepwise advancement of bone translocation. **F, G,H** Images taken at 1, 3, and 6 months post-docking, depicting progressive bone consolidation. **I, J** Anteroposterior and lateral radiographs demonstrating significant bone regeneration and proper alignment. **K, L,M** Functional assessment of the patient, exhibiting a restored range of motion and muscle strength during standing, squatting, and side bending movements, indicative of a successful trifocal bone transport process

**Fig. 2:**
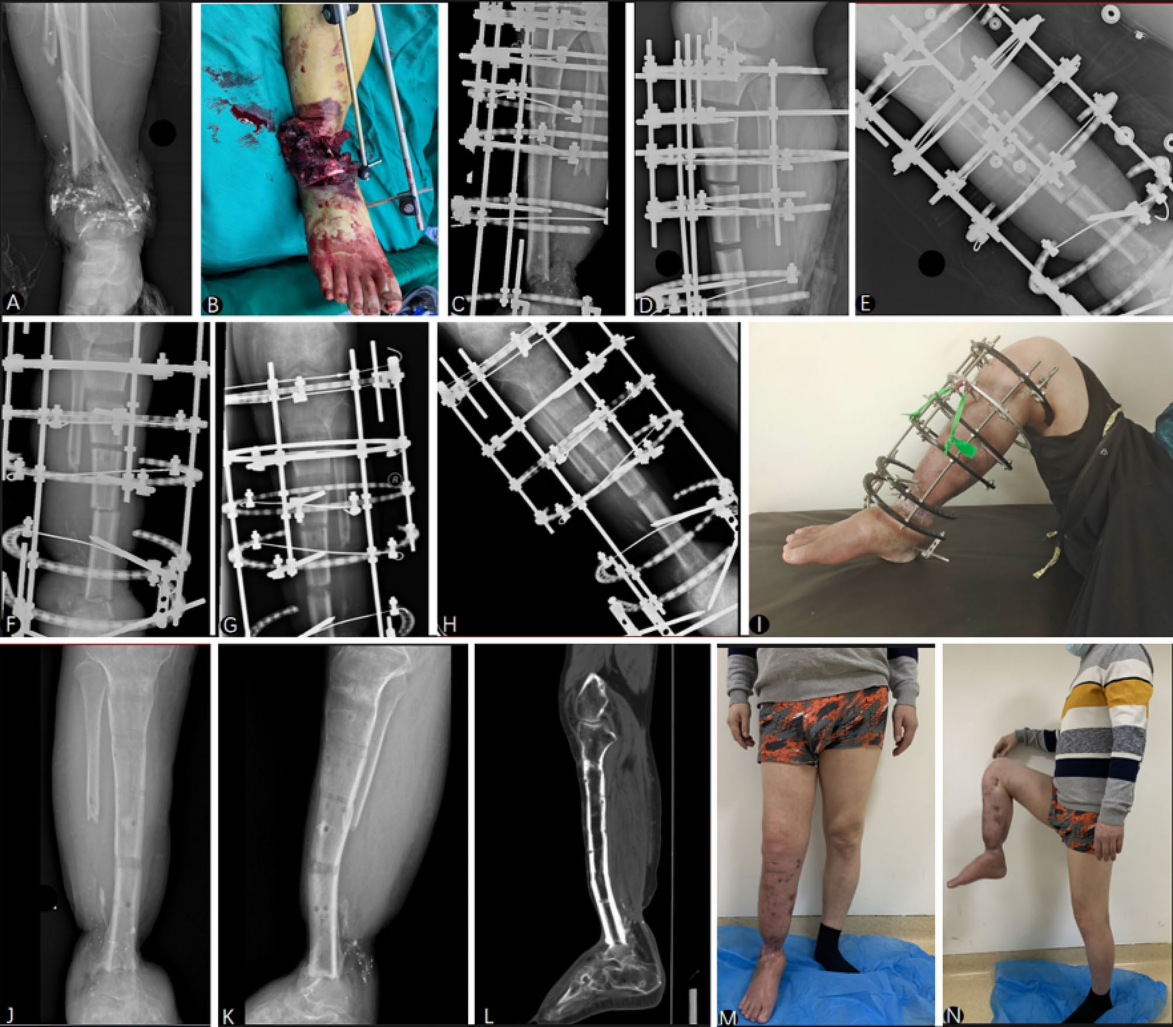
**A** Initial anteroposterior radiograph of a 38-year-old male outlining the preoperative condition. **B** Intraoperative image showcasing the severity of tissue damage. **C** Immediate postoperative anteroposterior radiographs showing the initial phase of external fixation. **D, E,F, G,H** Progressive radiographic sequence highlighting the stages of pentafocal bone transport, showcasing the initial external fixation, the progressive bone transport, and the stages of bone consolidation. **I** Clinical photograph of the external fixator in place, demonstrating the patient’s limb alignment during treatment. **J, K,L** Anteroposterior and lateral radiographs after the completion of the bone transport, indicating the new bone growth and alignment. **M, N** Final clinical photographs of the patient demonstrating the range of motion and improved functional capacity of the lower limb at the last follow-up

After conducting 1:1 propensity score matching to ensure comparability between both groups, our study included a total of 20 patients, with 10 patients undergoing PBT and an equal number of 10 patients selected from the 36 who underwent TBT. The average defect size was 6.86 ± 0.60 cm (range, 6.1 to 7.7 cm) in the TBT group and 7.17 ± 0.75 cm (range, 6.1 to 8.2 cm) in the PBT group. The follow-up period for all patients was at least two years, with an average duration of 31.4 ± 6.8 months (range, 24 to 44 months). There were no significant differences between the two groups in terms of age, gender, affected side, defect size, previous operation time, and follow-up time (*P* > 0.05), as detailed in Table 1. Typical cases are shown in Figs. 1 and 2.

**Table 1:** Comparison of the demographic and preoperative baseline data

Parameter	Total	TBT group	PBT group	*P*-value
Mean age (years)	40.9 ± 9.8	41.0 ± 9.8	40.7 ± 10.3	0.947
Gender (male/female)	16/4	7/3	9/1	0.582
Affected side (left/right)	14/6	8/2	6/4	0.628
Mean defect size (cm)	7.02 ± 0.68	6.86 ± 0.60	7.17 ± 0.75	0.320
Mean previous operation time (n)	2.1 ± 1.0	2.0 ± 1.1	2.2 ± 1.0	0.673
Mean follow-up period (months)	31.4 ± 6.8	33.1 ± 7.2	29.6 ± 6.2	0.260

**TBT** trifocal bone transport, **PBT** pentafocal bone transport

### Postoperative outcomes

In terms of surgical outcomes, the TBT group exhibited a longer average EFT of 297.3 ± 14.3 days (range, 276 to 320 days), in contrast to the PBT group, which had a notably shorter EFT of 130.9 ± 16.0 days (range, 115 to 159 days). Similarly, the EFI was significantly lower in the PBT group. The differences in both EFT and EFI between the TBT and PBT groups were statistically significant (*P* < 0.05), as shown in Table 2.

**Table 2:** Comparison of the postoperative outcomes

	TBT group	PBT group	*P*-value
Mean EFT (days)	297.3 ± 14.3	130.9 ± 16.0	< 0.001
Mean EFI (days/cm)	35.86 ± 3.69	20.67 ± 2.75	< 0.001

**TBT** trifocal bone transport, **PBT** pentafocal bone transport, **EFT** external fixation time, **EFI** external fixation index

### Bone and functional outcomes

The ASAMI criteria were utilized for evaluating both bone union and functional outcomes [17]. The detailed results of this assessment are tabulated in Table 3. In the TBT group, the ASAMI score indicated that 30% of patients achieved ‘excellent’ bone outcomes, while 30% achieved ‘good’ outcomes. Furthermore, the treatment with TBT patients achieved an ‘excellent’ functional outcome in 40% of cases, while a ‘good’ outcome was observed in 30%. Similarly, within the PBT group, bone outcomes categorized as ‘excellent’ and ‘good’ were observed in 50% and 20% of the cases, respectively. Functional outcomes were observed, with 20% and 40% of patients attaining ‘excellent’ and ‘good’ evaluations, respectively. Despite the variances in the distribution of ‘excellent’ and ‘good’ outcomes, overall, both groups demonstrated satisfactory bone and functional recovery. The absence of significant differences between the groups in achieving these outcomes underscores superior bone regeneration and union, suggesting a high level of postoperative mobility and a lower incidence of residual functional impairment.

**Table 3:** Comparison of the bone and functional results according ASAMI classification

Outcomes	Treament	Numbers/Percentage				*P*-value
		Excellent	Good	Fair	Poor	
Bone results	TBT	3 (30%)	3 (30%)	2 (20%)	2 (20%)	0.406
	PBT	5 (50%)	2 (20%)	2 (20%)	1 (10%)	
Functional results	TBT	4 (40%)	3 (30%)	2 (20%)	1 (10%)	0.453
	PBT	2 (20%)	4 (40%)	3 (30%)	1 (10%)	

**TBT** trifocal bone transport, **PBT** pentafocal bone transport

### Complications

The complications, assessed using the Paley classification, are methodically tabulated in Table 4 for comprehensive review [26]. In the TBT cohort, a total of 27 complications were documented. This included 17 instances categorized as ‘problems’, 6 cases classified as ‘obstacles’, and 4 cases classified as ‘true complications’. Among the most prevalent complications were muscle contraction, noted in 60% of the cases, and pin site infection, observed in 50% of the patients. Other notable complications included axial deviation (40%), delayed consolidation (40%), joint stiffness (40%), and delayed union or nonunion (30%). Additionally, complications classified under “other” in this cohort included rare and potentially unexpected issues such as nerve and vascular injury, joint dislocation, refracture at the regeneration site or docking, and recurrence of osteomyelitis, which collectively underscore the complexity of managing such severe cases. The PBT group, on the other hand, encountered 22 complications in total. This encompassed 12 ‘problems’, 8 ‘obstacles’, and 2 ‘true complications’. Notable complications in this group included pin site infection, occurring in a significant 100% of cases, axial deviation, seen in 40% of patients, and muscle contraction (30%), which was also common.

**Table 4:** Comparison of the complications accoding Paley criteria

Parameter	Treament	Problems	Obstacles	True Complications	Total	*P*-value
Muscle contraction	TBT	3	1	2	6	0.370
	PBT	2	1	0	3	
Axial deviation	TBT	2	1	1	4	1.000
	PBT	1	2	1	4	
Pin problems	TBT	3	2	0	5	0.033
	PBT	6	3	1	10	
Delayed consolidation	TBT	4	0	0	4	0.303
	PBT	1	0	0	1	
Delayed union or nonunion	TBT	2	1	0	3	1.000
	PBT	0	2	0	2	
Joint stiffness	TBT	3	1	0	4	0.628
	PBT	2	0	0	2	
Other	TBT	0	0	1	1	1.000
	PBT	0	0	0	0	
Total		29	14	6		

**TBT** trifocal bone transport, **PBT** pentafocal bone transport

## Discussion

The present comparative analysis of TBT and PBT in the management of distal tibial defects > 6 cm resulting from posttraumatic osteomyelitis provides a comprehensive understanding of the complexities and effectiveness of these advanced orthopedic interventions. The results of our study highlight the complexities of these methods and their impact on patient outcomes, especially in terms of potentially shortening treatment times and managing complications.

The Ilizarov bone transport technique has garnered significant recognition due to its efficacy in promoting bone regeneration and addressing substantial osseous gaps. However, one major challenge related to this method is the prolonged period of external fixation, which frequently results in a variety of issues for patients [17–24]. Addressing such issues is crucial for advancing the field of limb reconstruction and improving patient outcomes. This necessity paves the way for exploring modifications to the traditional Ilizarov technique or integrating adjunctive treatment modalities, aiming to expedite the bone healing process while minimizing the associated complications of prolonged external fixation.

The proposal of employing higher distraction rates and increasing daily distraction quantity has been posited as a strategy to expedite bone regeneration. While this approach might offer the advantage of shortened treatment times, it also bears risks such as suboptimal bone formation, potential for nonunion, or increased discomfort for patients [27, 28]. Thus, the application of higher distraction rates must be judiciously considered to avoid compromising treatment quality.

The integration of the external fixator with internal fixation methods such as plate fixation has been explored as a means to stabilize the bone segments more rigidly by Gupta et al. [29], potentially allowing for earlier removal of the external fixator. Other studies have also performed the reconstruction of long bone defects using bone transport over an intramedullary nail. These studies have reported satisfactory clinical outcomes, as they have successfully reduced the duration of external fixator usage [30, 31]. While this approach could theoretically reduce the duration of external fixation and associated complications such as pin site infections and joint stiffness, it also brings challenges. These include the risk of infection at the site of internal fixation, stress shielding, and difficulties in achieving proper alignment and stabilization, which are crucial for successful bone healing.

Building upon these insights, the concept of multifocal bone transport has emerged as a pivotal advancement, offering a nuanced approach to managing extensive bone defects while potentially mitigating the challenges associated with prolonged use of traditional fixation methods. Paley et al. [18] reported on the treatment of 19 patients with tibial defects using either single- or double-level bone transport, noting a mean EFT of 16 months. Similarly, Borzunov et al. [17] demonstrated the effectiveness of multifocal bone transport with multilevel osteotomy in decreasing EFT and achieving favorable bone outcomes when compared to bifocal bone transport. Expanding upon this foundation, Catagni et al. [22] delved into a comparative analysis between trifocal and bifocal bone transport for tibial defects. Their study revealed the efficiency of trifocal methods in significantly reducing the duration required for tibial reconstruction while simultaneously decreasing the likelihood of additional surgical interventions. To further reinforce the advantages of multifocal techniques, Yushan et al. [19] compared trifocal bone transport with bifocal methods in the context of extensive tibial defects. Their research underscored the former’s ability to considerably reduce the time required for bone repair and mitigate the challenges associated with prolonged treatment periods. Additionally, another comparative study highlighted that the use of tetrafocal and pentafocal bone transport could effectively shorten the distraction period, accelerate regeneration, and minimize complications [25].

The concept of multifocal bone transport, which was particularly prominent in our PBT group, is a testament to the evolving complexity of reconstructive bone surgery. By employing multiple corticotomies, PBT allows for a broader scope of bone regeneration than TBT, making it suitable for more extensive defects. This approach, while innovative, introduces additional complexities in surgical management and postoperative care. The challenges associated with managing multiple regeneration zones must be balanced against the potential benefits of this approach, especially in terms of reduced treatment duration and improved functional outcomes. Therefore, it is recommended that during the period of segmental bone transportation, until the complete contact of the docking site is reached, patients should stay in the hospital and receive daily guidance from a professionally trained Ilizarov surgeon to minimize the risk of complications. It is imperative to acknowledge that multifocal bone transport via the Ilizarov technique is a specialized and intricate surgical procedure that demands a considerable learning curve, in the hands of an inexperienced surgeon, the outcomes may not be optimal. In addition, the precise positioning of the osteotomy site is crucial. The primary blood supply to long bones, provided by nutrient arteries, plays a crucial role during growth periods and the ossification process in fracture healing. Recognizing the importance of maintaining adequate blood supply for vascularized bone and fostering neovascularization during bone regeneration, it becomes essential to select an osteotomy level that carefully avoids disrupting the nutrient foramina. Notably, the bulk of these foramina in the tibia are typically situated in the two-fifths portion of the bone [32].

One crucial analysis component involved comparing the EFT and EFI between the TBT and PBT groups. The PBT group exhibited a shorter average EFT and EFI, indicating a potential advantage in terms of shorter external fixation times and potentially more efficient bone regeneration compared to the TBT group. This finding is particularly significant as it indicates that despite the increased complexity of PBT, it may offer an expedited path to recovery. However, it’s essential to balance this benefit against the increased risks associated with more intricate surgery, such as the increased possibility of complications. Our study highlighted a significant increase in the rate of pin track infections in the PBT group compared to the TBT group, a finding that underscores the inherent risks associated with more complex surgical procedures. Managing a greater number of pin sites in multifocal procedures inherently elevates the risk of infection. Additionally, the occurrence of other complications, such as muscle contraction, joint stiffness, and delayed union or nonunion, was similar across the groups. This indicates that certain complications are intrinsic to the nature of extensive bone transport procedures, regardless of the method employed. It is essential to mention that the utilization of the Ilizarov technique for PBT may effectively reduce tensional stress on the surrounding soft tissues by distributing it across multiple segments, as opposed to TBT. This approach has the potential to greatly benefit patients by effectively mitigating postoperative pain commonly associated with soft tissue tension during extensive bone transportation.

While the present study provides insights, it is not without limitations. The retrospective nature and the relatively small sample size may affect generalizability of the findings. Future research should include larger, prospective studies with longer follow-up periods to validate and extend our findings. Additionally, exploring the long-term impact of these techniques on patients’ quality of life and functional status will be crucial for fully understanding their efficacy.

## Conclusion

In summary, both TBT and PBT have emerged as effective strategies for managing complex segmental tibial defects. While PBT potentially results in more efficient bone regeneration, it also carries a greater risk of complications such as pin site infections. It is important to note that while both TBT and PBT can yield satisfactory outcomes, they are specialist surgical techniques that take a significant amount of expertise to master. The PBT procedure necessitates the performance of two extra osteotomies and a precise transport time-schedule. Consequently, the treatment outcomes can be risky and unsatisfactory in an inexperienced surgeon’s hands or without prudent patient selection.

## Data Availability

Data is provided within the manuscript.
